# Adaptive Interacting Multiple Model Algorithm Based on Information-Weighted Consensus for Maneuvering Target Tracking

**DOI:** 10.3390/s18072012

**Published:** 2018-06-22

**Authors:** Ziran Ding, Yu Liu, Jun Liu, Kaimin Yu, Yuanyang You, Peiliang Jing, You He

**Affiliations:** 1 Research Institute of Information Fusion, Naval Aviation University, Yantai 264001, China; DZR19931201@163.com (Z.D.); heyou_f@126.com (Y.H.); 2 School of Electronic and Information Engineering, Beihang University, Beijing 100191, China; 3 The First Training Base, Naval Aviation University, Huludao 125001, China; dongyongdi725@163.com; 4 The Second Training Base, Naval Aviation University, Changzhi 046000, China; yyyy-abc@163.com; 5 China Ordnance Test Center, Huayin 714200, China; jingpeiliang@nudt.edu.cn

**Keywords:** state estimation, maneuvering target tracking, multiple sensor fusion, consensus, interacting multiple model

## Abstract

Networked multiple sensors are used to solve the problem of maneuvering target tracking. To avoid the linearization of nonlinear dynamic functions, and to obtain more accurate estimates for maneuvering targets, a novel adaptive information-weighted consensus filter for maneuvering target tracking is proposed. The pseudo measurement matrix is computed with unscented transform to utilize the information form of measurements, which is necessary for consensus iterations. To improve the maneuvering target tracking accuracy and get a unified estimation in each sensor node across the entire network, the adaptive current statistical model is exploited to update the estimate, and the information-weighted consensus protocol is applied among neighboring nodes for each dynamic model. Based on posterior probabilities of multiple models, the final estimate of each sensor is acquired with weighted combination of model-conditioned estimates. Experimental results illustrate the superior performance of the proposed algorithm with respect tracking accuracy and agreement of estimates in the whole network.

## 1. Introduction

The maneuvering target tracking problem has drawn immense attention for many years in areas such as aircraft surveillance, radar tracking, and road vehicle navigation [1,2]. In the military field, to avoid being attacked, most of the enemy’s targets have strong maneuvers. In this situation, traditional tracking algorithms are not as effective as they usually are. Once the targets maneuver, they will be out of detection soon. Maneuvering target tracking has become a common situation in the military field, the research of which is of great significance. However, as the target’s maneuverability and the anti-tracking awareness of fighters are increased, tracking maneuvering targets becomes increasingly difficult.

In maneuvering target tracking, the uncertainty of the target motion model leads to large errors for tracking results. To solve the problem, a variety of methods, including the white noise model with adjustable level, the variable dimension model, input estimation model, Singer model, Jerk model, multiple model and interacting multiple model (IMM), have been proposed. Among them, IMM [3] has won great popularity. In maneuvering target tracking, the IMM algorithm has been shown to be one of the most cost-effective estimation schemes [4]. In IMM, the model set includes several models to deal with the varying characteristics of target motion, as it is assumed that the target trajectory evolves according to some predetermined models. However, multiple models sometimes cannot fully contain the target’s motion, and excessive numbers of models will generate larger calculations. On the basis of IMM, the interacting multiple adaptive model (IMAM) [5] uses two adaptive current statistical models to form a model set. The IMAM has better adaptability for model uncertainty, and it can achieve better accuracy than IMM when the model set of IMM cannot contain the motion of the target.

The standard extended Kalman filter (EKF) has become the method of choice in a number of nonlinear estimations. The EKF uses Taylor series expansions to handle nonlinear states and observation transition functions. However, when the system is highly nonlinear, the linearization scheme will cause additional linearization errors [6]. The unscented Kalman filter (UKF) approximates the posterior probability distribution by a selected set of sigma points, which are propagated according to the unscented transformation [7]. The estimated mean and covariance are obtained by weighted sum of these unscented points. As for highly nonlinear systems, UKF is more accurate and less computational than EKF. The Particle Filter (PF) has a superior performance for solving nonlinear problem than EKF and UKF, and can even be applied to non-Gaussian problems [8]. However, it costs too much in computational resources.

Many studies focus on maneuvering target tracking under nonlinear system. Reference [9] uses the current statistical model to estimate the state of the maneuvering target, in which the adaptive process noise covariance is applied to reduce the influence of limited acceleration. To handle nonlinear systems, the current statistical model is based on UKF. Reference [10] proposed an efficient IMM-UKF algorithm. Apart from the advantages of IMM and UKF, the cubature-principle-assisted IMM method can deal with the non-Gaussian problem, and the new adaptive UKF algorithm addresses sensor faults. Based on PF, Reference [11,12] performed a very similar study. They both fused the information of multiple predefined models to obtain a more accurate result, and utilized PF to estimate the target. The difference is that [11] allocates particles on the basis of posterior probability of each model, while [12] achieves allocation according to predictive power. The allocation of particles can provide more resources to the model which is closer to the true target motion, and then this model can obtain a more accurate estimate at the next time. Reference [13] proposed Particle Learning (PL), showing that PL outperforms existing PF. In PL, particles are used to approximate joint posterior distribution of the state and static parameters, so they can describe the feature of target more accurately. Due to the large number of particles used in the computing process, the defect of these particle methods is their heavy calculation, which would be particularly obvious in the multi-sensor system. References [10,11,12,13] are only based on a single sensor, so they cannot utilize the superiority of sensor networks which fuse the information of multiple sensors.

In many tracking scenarios, multiple sensors are used to provide more complementary measurement information and higher tolerance about individual sensor failure. There are three main kinds of multi-sensor data fusion architectures: centralized, hierarchical distributed and distributed [14]. Centralized architecture is the traditional architecture in which measurements from all sensors are sent to the fusion center. The optimal estimation is obtained by processing and fusing the complete measurement sets of the whole system [15]. However, the computation and communication burden imposed on the fusion center are severe, only powerful equipment can accomplish this task. On the other hand, a center node’s failure results in failure of the whole system. In a hierarchical distributed architecture, multiple sensors are managed in a hierarchy. The low-level sensors handle measurements and send results to higher level sensors. The higher-level sensors only need to transmit processing results; thus, they have a lower computation burden and require less communication bandwidth. The distributed architecture has no fusion center. Each sensor node only exchanges local state estimation information with its neighbor nodes. This architecture requires less communication bandwidth and has a lower computation burden in each sensor, and it is strongly tolerant for single sensor failure [16,17]. However, the information from neighbor sensors is limited, which will cause a decline in the estimation accuracy. Through the consensus iterations, the estimated value of each sensor gradually tends to the global optimal estimation [18,19], which ensures a consensual estimate of the situation and improves estimation accuracy for each sensor [20].

To solve the model uncertainty of the target by the input estimation technique, an adaptive particle filter for maneuvering target tracking [21] is proposed. The multiplicative measurement model is used to deal with the measurement noise (related to state) in the sensor network. In addition, all measurements of sensors are sent to the fusion center. In sensor networks, IMM is still a great algorithm to deal with the maneuver of the target [22]. The likelihoods of models are communicated to the central-level tracker along with the measurements, so the effects of sensor biases can be reduced. The distributed state estimation with model uncertainty is considered by [23]. The distributed mixture Kalman filter is an effective method to handle nonlinear system and maneuvering target. Based on a distributed architecture, average consensus is executed to improve the accuracy of all sensors.

In this paper, an adaptive interacting multiple model algorithm based on information-weighted consensus (IMAM-UICF) is proposed. This algorithm further improves the estimation accuracy of tracking maneuvering target on the basis of IMAM, and the consensus filter is the key to the improvement. Each sensor in the network uses IMAM to get a more realistic model hypothesis of target motion. The information filter can estimate the target of a nonlinear system by embedding the unscented transformation. Then each sensor node maintains a local estimate and interacts with neighbor nodes. After receiving information from neighbor nodes, each node corrects its local estimate according to the consensus protocol. The algorithm can accomplish the maneuvering target tracking task under nonlinear systems with high estimation accuracy.

The remainder of this paper is organized as follows. The sensor communication topology and system model are defined in Section 2. The nonlinear interacting multiple adaptive model is described in Section 3. Our algorithm IMAM-UICF is proposed in Section 4. Experimental evaluation in Section 5 demonstrates the effectiveness of the proposed algorithm and comparisons with other methods. Finally, the conclusions are provided in Section 6.

## 2. Problem Formulation

### 2.1. Communication Network

The communication network topology between multiple sensors is represented by the adjacency matrix based on graph theory. A graph is defined as G=(N,E), where N={1,2,⋯,n} is the set of sensor nodes and E⊆N×N is a set of edges in graph *G*. The edges represent the available communication channels between two nodes. $N_{i}=\{j\in N,(j,i)\in E\}$ denotes the set of neighbor sensors that can communicate with sensor i.

**Figure 1:**
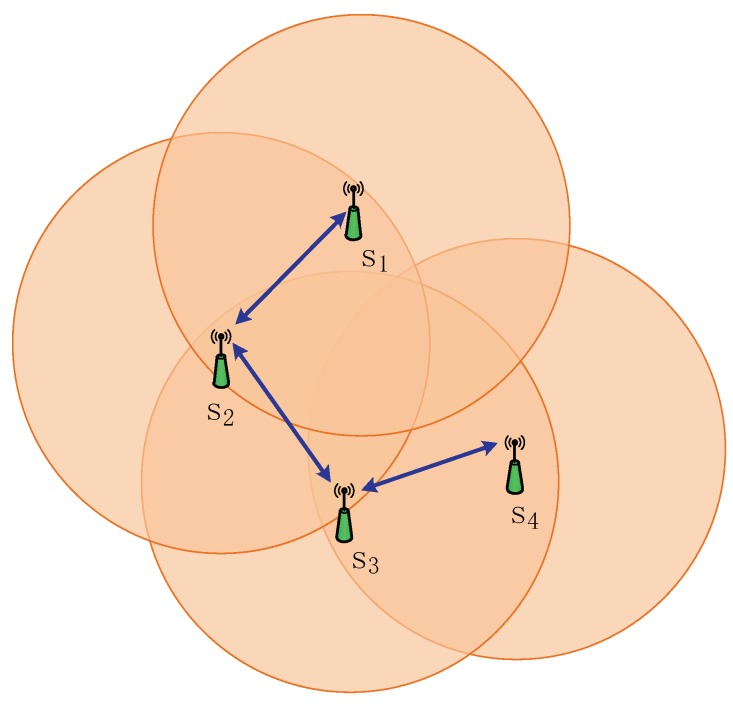
Communication network. $s_{i}$ represents the number of the sensor. The dotted line denotes the communication range of the sensor. The blue arrow represents the communication between the sensors.

Adjacency matrix $A={\left[a_{ij}\right]}_{i,j=1}^{n}$ is defined as $a_{ij}=\{\begin{array}{cc} 1 & if(j,i)\in E\\ 0 & otherwise \end{array}$, where $a_{ij}=1$ indicates that there is a communication link between node i and node j, and $a_{ij}=0$ indicates that node i cannot communicate with node j. In Figure 1, for example, there are 4 sensors in the network.

The corresponding network topology can be denoted by the following adjacency matrix:

(1) $$A=\left[\begin{array}{cccc} 0 & 1 & 0 & 0\\ 1 & 0 & 1 & 0\\ 0 & 1 & 0 & 1\\ 0 & 0 & 1 & 0 \end{array}\right]$$

### 2.2. System Modeling

The state variable method is a valuable method to describe the dynamic system. The relationship between input and output of the system is described in the time domain using the state transfer model and output observation model. The input can be described by the state equation. The state equation consists of a determined time function and a stochastic process, and the stochastic process represents unpredictable noise. The output is a function of the state vector. The output is usually disturbed by the stochastic observation error and can be described by the measurement equation.

State equation:

(2) $$x(k)=f(x(k-1))+G\bar{a}(k-1)+w(k-1)$$

where x(k) represents the estimated state of target at the filtering time k, and the process noise is subject to w(k−1)~N(0,Q(k−1)). f(⋅) denotes state transfer function and G denotes input control matrix, $\bar{a}(k-1)$ is maneuvering acceleration mean.

Measurement equation:

(3) z(k)=h(x(k))+r(k)

where z(k) represents the measurement of sensor, the measurement noise r(k) is subject to r(k)~N(0,R(k)), and h(⋅) denotes the measurement transfer function.

When networked multiple sensors observe a target, they get different measurements due to their different observation abilities. Assuming that there are n sensors, the measurement in Equation (3) should be rewritten as:

(4) $$z_{i}(k)=h_{i}(x(k))+r_{i}(k),i=1,2,\cdots ,n$$

How to fuse multiple measurements from different sensors to obtain the global optimal estimation is the main problem that consensus-based distributed filtering algorithms take into consideration.

When a maneuvering target is tracked, the motion state of the target is changeable, that is, there are multiple possible state transfer functions $f^{r}(\cdot ),r=1,2,\cdots ,m$.

Then the state equation of the target should be rewritten as:

(5) $$x(k)=f^{r}(x(k-1))+G^{r}\bar{a}(k-1)+w^{r}(k-1),r=1,2,\cdots ,m$$

where r=1,2,⋯,m denotes the $r_{th}$ model, and m is the number of models.

## 3. Nonlinear Interacting Multiple Adaptive Model

### 3.1. Interacting Multiple Adaptive Model

IMAM [5] is an interacting multiple model method that selects two ACS models with different maneuver frequencies to form a model set.

In the current statistical (CS) model, the fixed maximum acceleration is one reason that results in the increase of the model’s error after the target maneuvers. Adaptive current statistical (ACS) model is a model that can adaptively adjust the maximum acceleration. It can improve the CS model’s ability to respond to maneuvering targets, and ensure that the CS model has an accurate description of maneuvering targets.

#### 3.1.1. Current Statistical Model

In the IMM [24], the system model is composed of multiple motion models. Target state is respectively estimated by each model in the tracking process, and the probability of each model is constantly adjusted at the same time. Considering the probability of each mode as the corresponding weight, the final estimate of IMM is the weighted sum of the estimated value of each model.

***Model Interaction***

The transition probability of the moving state of the target is defined as

(6) $$P=\left[\begin{array}{cccc} P(1,1) & P(1,2) & \cdots & P(1,m)\\ P(2,1) & P(2,2) & \cdots & P(2,m)\\ \vdots & \vdots & \ddots & \vdots \\ P(m,1) & P(m,2) & \cdots & P(m,m) \end{array}\right]$$

where m is the number of models in the model set.

$u_{j|r}(k-1|k-1)$ is the probability that the model r is converted from the model j.

(7) $$u_{j|r}(k-1|k-1)=\frac{1}{\bar{C_{j}}}P(j,r)u_{r}(k-1)$$

where $u_{r}(k-1)$ is the probability of model r at time k−1, $\bar{C_{j}}=\sum_{r=1}^{m}P(j,r)u_{r}(k-1)$.

$x_{}^{or}(k-1|k-1)$ is the state estimation of model r at time k−1, $P_{}^{or}(k-1|k-1)$ is the corresponding state covariance. The input of the mode r after the interactive calculation is as follows

(8) $$x_{}^{or}(k-1|k-1)=\sum_{j=1}^{m}x_{j}(k-1|k-1)u_{j|r}(k-1|k-1)$$

(9) $$\begin{array}{l} P_{}^{or}(k-1|k-1)=\sum_{j=1}^{m}\{P_{j}(k-1|k-1)+[x_{j}(k-1|k-1)-x_{}^{or}(k-1|k-1)]\\ {\left[x_{j}(k-1|k-1)-x_{}^{or}(k-1|k-1)\right]}^{T}\}u_{j|r}(k-1|k-1) \end{array}$$

***Model-Conditioned Filtering***

Use state vector $x_{}^{or}(k-1|k-1)$, covariance $P_{}^{or}(k-1|k-1)$ and measurement z(k) as the input of model r at time k. Each model obtains output $x_{r}(k|k)$ and $P_{r}(k|k)$ based on its own model and input.

***Model-Conditioned Filtering***

Assuming that the innovation $\nu_{r}(k)$ obeys the Gauss distribution, the likelihood function is $\Lambda_{r}(k)$.

(10) $$\Lambda_{r}(k)=\frac{1}{\sqrt{\left|2\pi S_{r}(k)\right|}}\exp[-\frac{1}{2}{\left(\nu_{r}(k)\right)}^{T}{\left(S_{r}(k)\right)}^{-1}\nu_{r}(k)]$$

where $\nu_{r}(k)=z(k)-H_{r}(k)x_{r}(k|k-1)$, and $S_{r}(k)$ is its covariance, $S_{r}(k)=H_{r}(k)P_{r}(k|k-1){\left(H_{r}(k)\right)}^{T}+R_{}^{-1}$.

The posteriori model probability of model r is calculated as follows:

(11) $$u_{r}(k)=\frac{1}{C}\Lambda_{r}(k)\bar{C_{r}}$$

where $C=\sum_{r=1}^{m}\Lambda_{r}(k)\bar{C_{r}}$.

***State Combination***

The combined state x(k|k) and its covariance P(k|k) are calculated as

(12) $$x(k|k)=\sum_{r=1}^{m}x_{r}(k|k)u_{r}(k)$$

(13) $$P(k|k)=\sum_{r=1}^{m}u_{r}(k)\{P_{r}(k|k)+[x_{r}(k|k)-x(k|k)]\cdot {\left[x_{r}(k|k)-x(k|k)\right]}^{T}\}$$

#### 3.1.2. Current Statistical Model

In the current statistical model, the process noise covariance matrix is time-varying. For example, there is an estimate of one-dimensional coordinate $x={\left[x\;\dot{x}\;\ddot{x}\right]}^{T}$, its process noise covariance matrix is

(14) $$Q(k)=2\alpha \sigma_{a}^{2}(k)q$$

where α is the maneuver frequency, q is a constant matrix related to α and the sampling period T, and its expression can be referred to literature [25].

$\sigma_{a}^{2}(k)$ represents the variance of the maneuvering acceleration at time k, and it is adaptively adjusted at each moment. Its value is

(15) $$\sigma_{a}^{2}(k)=\left\{ \begin{array}{l} \frac{4-\pi }{\pi }{\left[a_{\max}-\bar{a}(k)\right]}^{2},\bar{a}(k)>0\\ \frac{4-\pi }{\pi }{\left[a_{-\max}-\bar{a}(k)\right]}^{2},\bar{a}(k)<0 \end{array}\right.$$

where $\bar{a}(k)=\hat{\ddot{x}}(k|k-1)$. i is the maximum acceleration. In the traditional current statistical model, $a_{\max}$ is a fixed value given in advance according to experience. 

#### 3.1.3. Adaptive Current Statistical Model

Structure membership function [5]:

(16) $$u_{\delta }(k)=\left\{ \begin{array}{l} 1-e^{-\nu {\left(k\right)}^{2}/(R_{1}\cdot c)},\left|\nu (k)\right|<3R_{1}\\ 1-e^{-\nu {\left(k\right)}^{4}/(R_{1}\cdot c)},\left|\nu (k)\right|\geq 3R_{1} \end{array}\right.$$

where *ν*(*k*) represents the innovation at time k, $R_{1}$ denotes the position measurement noise, and c denotes the position estimation covariance

In the ACS model, the maximum acceleration $a_{\max}$ adaptively changes according to the following equation:

(17) $$a_{\max}=\bar{a}(k)+2u_{\delta }(k)\cdot \left|\nu (k)\right|$$

The ACS model adjusts the maximum acceleration $a_{\max}$ at each moment according to the innovation, making it include the true acceleration of the target motion as much as possible. This method improves the response of the CS to the maneuver, which results in a better tracking effect. Selecting two ASC models, IMAM quickly adapts to the mutation of the target movement. Comparing to the traditional IMM, IMAM has a better performance for maneuvering target tracking.

### 3.2. Nonlinear Interacting Multiple Adaptive Model

In our algorithm, the Unscented Transformation (UT) of UKF [26] is chosen to deal with nonlinear problems. In nonlinear systems, UT transfers statistical characteristics through a set of random sampling points, so the prediction can be done. The predicted state x(k|k−1), predicted covariance P(k|k−1) and predicted measurement z(k|k−1) can be obtained by using the prediction of sigma points and corresponding weight. The details of UT are explained below.

In the unscented transformation, $\left(2n_{x}+1\right)$ sigma points are usually selected for the target state

(18) $$\{\begin{array}{ll} \xi_{0}(k-1|k-1)=x_{}^{or}(k-1|k-1) & p=0\\ \xi_{p}(k-1|k-1)=x_{}^{or}(k-1|k-1)\;+{\left(\sqrt{(n_{x}+\kappa )P_{}^{or}(k-1|k-1)\;}\right)}_{p} & p=1,\cdots ,n_{x}\\ \xi_{p+n_{x}}(k-1|k-1)=x_{}^{or}(k-1|k-1)-{\left(\sqrt{\left(n_{x}+\kappa )P_{}^{or}(k-1|k-1\right)}\right)}_{p} & p=1,\cdots ,n_{x} \end{array}$$

where κ is a scale parameter, usually $\kappa =n_{x}(\tau^{2}-1)$, the range of the parameter τ is 0.0001≤τ≤1; ${\left(\sqrt{(n_{x}+\kappa )P_{}^{or}(k-1|k-1)\;}\right)}_{p}$ is the $p_{th}$ row or the $p_{th}$ column of the root mean square matrix $\sqrt{\left(n_{x}+\kappa )P_{}^{or}(k-1|k-1\right)}$; $n_{x}$ is the dimension of the state vector.

These sigma points are symmetric distributions of the mean target state, and they can describe the random quantity of the Gauss distribution well. The weights corresponding to sigma points are

(19) $$W_{h}=\{\begin{array}{ll} \frac{\kappa }{\left(n_{x}+\kappa \right)} & h=0\\ \frac{1}{2(n_{x}+\kappa )} & h=1,2,\cdots ,2n_{x} \end{array}$$

A set of precise selected sigma points are transformed through the known nonlinear function. These points are used to transfer the statistical characteristics of random quantities. These characteristics are the probability distribution’s statistical moments: mean and covariance.

The prediction of state sigma points are

(20) $$\xi_{h}(k|k-1)=f^{r}\left(\xi_{h}(k-1|k-1)\right)$$

According to the nonlinear function propagation method of the unscented transformation, the predicted state x(k|k−1) and the predicted covariance of state P(k|k−1) can be obtained by using the prediction of state sigma points and corresponding weight

(21) $$x(k|k-1)=\sum_{h=0}^{2n_{x}}W_{h}\xi_{h}(k|k-1)+G^{r}\bar{a}(k-1)$$

(22) $$P(k|k-1)=\sum_{h=0}^{2n_{x}}W_{h}\Delta x_{h}(k|k-1)\Delta x_{h}^{T}(k|k-1)+Q^{r}(k-1)$$

where $\Delta x_{h}(k|k-1)=\xi_{h}(k|k-1)-x(k|k-1)$.

The prediction of measurement sigma points are $\varsigma_{h}(k|k-1)=h(k,\xi_{h}(k|k-1))$.

The weighted sum of the predicted measurement sigma points is the predicted measurement

(23) $$z(k|k-1)=\sum_{h=0}^{2n_{x}}W_{h}\varsigma_{h}(k|k-1)$$

The cross-correlation covariance of state and measurement is

(24) $$P_{}^{xz}(k|k-1)=\sum_{h=0}^{2n_{x}}W_{h}\Delta x_{h}(k|k-1)\Delta z_{h}^{T}$$

where $\Delta z_{h}=\varsigma_{h}(k|k-1)-z(k|k-1)$.

The research in [20] shows that, compared to common Kalman filtering, the consensus protocol can play a better role in Information Filtering (IF). Therefore, IF is selected as the basic filtering method of every model in the model set. In information filtering, update of the information matrix and information vector are based on the linear measurement transfer matrix. In order to update information in a nonlinear system, a pseudo measurement matrix is defined [27]. According to $P_{}^{xz}(k|k-1)=P(k|k-1)H_{}^{T}(k)$, we get $H_{}^{T}(k)={\left(P(k|k-1)\right)}^{-1}P_{}^{xz}(k|k-1)$.

Then the update of information matrix and information vector [20] are

(25) $$Y=\frac{1}{n}{\left(P(k|k-1)\right)}^{-1}+H_{}^{T}(k)R_{}^{-1}H(k)$$

(26) $$y=\frac{1}{n}{\left(P(k|k-1)\right)}^{-1}x(k|k-1)+H_{}^{T}(k)R_{}^{-1}(H(k)x(k|k-1)+z(k)-z(k|k-1))$$

where n is the number of sensors in the sensor network.

In linear systems, the innovation $\nu (k)=z(k)-z(k|k-1)=x_{z}(k)-x_{z}(k|k-1)$ represents the difference between the measurement and the predicted measurement in the x direction. In Equation (16), the innovation is used to calculate membership function. Then we adaptively adjust the maximum acceleration in the x direction according to the membership function. However, in nonlinear systems, the innovation $\nu (k)=z(k)-z(k|k-1)=\left|\begin{array}{l} \rho_{z}(k)\\ \theta_{z}(k) \end{array}\right|-\left|\begin{array}{l} \rho_{z}(k|k-1)\\ \theta_{z}(k|k-1) \end{array}\right|$ cannot be used to calculate membership function. Through theoretical analysis and testing, we find that using $\left[\rho_{z}(k)-\rho_{z}(k|k-1)]\times \cos[\theta_{z}(k)-\theta_{z}(k|k-1)\right]$ instead of the innovation to calculate the membership function will return results with larger errors. In contrast, using $\rho_{z}(k)\times \cos\theta_{z}(k)-\rho_{z}(k|k-1)\times \cos\theta_{z}(k|k-1)$ instead of the innovation, we get results that describe the difference between the measurement and the predicted measurement more accurately.

Based on the above three methods, the proposed algorithm has the ability to track maneuvering targets in a nonlinear system. In addition, errors caused by nonlinear problems are controlled within a very small range.

## 4. Adaptive Interacting Multiple Model Algorithm Based on Information-Weighted Consensus

Considering the changeable moving state of a maneuvering target, we use the concept of IMAM to get a set of system models that are closer to the actual situation. This can compensate for the decline of tracking performance caused by the target maneuver. At the same time, considering the limited detection and survivability of single sensor, we build a multi-sensor network system to track the target. Multi-sensor data fusion can improve the tracking accuracy, and the distributed architecture ensures the robustness and flexibility of the system. The consensus protocol is combined with the distributed architecture, and it overcomes the shortage of distributed structure that improves the consensus of multiple sensors’ estimation situations and estimate accuracy [28].

### 4.1. Average Consensus

Average consensus is an effective method to fuse the information of multiple distributed sensors. It is the most popular consensus protocol and has many applications in filter algorithms. Average consensus makes the estimates converge to their mean. For example, there is a network of n nodes, and each node has an estimate value $a_{i}$. $a_{i}(0)=a_{i}$ is the initial value of each node, then runs the iterative formula for L times:

(27) $$a_{i}(l)=a_{i}(l-1)+e\sum_{s\in N_{i}}\left(a_{s}(l-1)-a_{i}(l-1)\right)$$

Before iteration l, each node sends its estimate value $a_{i}(l-1)$ to its neighbor nodes s ($s\in N_{i}$) and receives neighbor node estimate value $a_{s}(l-1)$. Then new estimate value $a_{i}(l)$ is calculated as Equation (27). After L iterations, the estimate values of all nodes converge to the average of their initial values $\frac{1}{n}\sum_{i=1}^{n}a_{i}(0)$. In addition, theoretically, when L→∞, all final value $a_{i}(L)$ is equal to $\frac{1}{n}\sum_{i=1}^{n}a_{i}(0)$. Based on all estimates $a_{i}$, $\frac{1}{n}\sum_{i=1}^{n}a_{i}(0)$ is the optimal estimate that nodes in the communication network could get. The final estimates of all nodes $a_{i}(L)$ are close to the mean $\frac{1}{n}\sum_{i=1}^{n}a_{i}(0)$, this is the reason they have low disagreement.

The value of consensus weight e has a limit $\left(0,\frac{1}{\Delta_{\max}}\right)$, where $\Delta_{\max}$ is the maximum degree of the communication network graph. In addition, each pair of nodes could have different weights, even the weights can be time-varying. So e should be replaced by $e_{i,s}(k)$. Between 0 and $\frac{1}{\Delta_{\max}}$, there are many options for its value. In our algorithm, we choose Metropolis weights as consensus weights. Metropolis weights can provide a fast convergence rate [29]. They also have good effect in distributed architecture, because nodes do not need to know the communication graph and even the number of nodes. Their values are

(28) $$e_{i,s}(k)=\{\begin{array}{cc} \frac{1}{1+\max\{d_{i}(k),d_{s}(k)\}} & \begin{array}{cc} if & s\in N_{i} \end{array}\\ 1-\sum_{s\in N_{i}}e_{i,s}(k) & \begin{array}{cc} if & i=s \end{array}\\ 0 & otherwise \end{array}$$

where $d_{i}(k)$ represents the number of sensor i’s neighbors at time k.

### 4.2. Interacting Multiple Adaptive Model-Unscented Information Consensus Filter

To state the proposed method more clearly, the algorithm flow of IMAM-UICF is illustrated in Algorithm 1.

**Algorithm 1.** IMAM-UICF for sensor i at time instant k.**Input: state**
$x_{i,r}(k-1|k-1)$
**and error covariance**
$P_{i,r}(k-1|k-1)$**, process noise covariance**
$Q^{r}(k-1)$**, hybrid probability**
$u_{i,j|r}(k-1|k-1)$
**, measurement**
$z_{i}(k)$**;**
**Output: state**
$x_{i,r}(k|k)$
**and error covariance**
$P_{i,r}(k|k)$
**, process noise covariance**
$Q^{r}(k)$
**, hybrid probability**
$u_{i,j|r}(k|k)$
**, combination state**
$x_{i}(k|k)$
**and error covariance**
$P_{i}(k|k)$
**;**
**Step** **1.** Interacting  $x_{i}^{or}(k-1|k-1)=\sum_{j=1}^{m}x_{i,j}(k-1|k-1)u_{i,j|r}(k-1|k-1)$ 
$\begin{array}{l} P_{i}^{or}(k-1|k-1)=\sum_{j=1}^{m}\{P_{i,j}(k-1|k-1)+[x_{i,j}(k-1|k-1)-x_{i}^{or}(k-1|k-1)]\\ {\left[x_{i,j}(k-1|k-1)-x_{i}^{or}(k-1|k-1)\right]}^{T}\}u_{i,j|r}(k-1|k-1) \end{array}$
**Step** **2.** Filtering
 (1) prediction (based on the UT)
  $x_{i,r}(k|k-1)=\sum_{h=0}^{2n_{x}}W_{h}\xi_{h}(k|k-1)+G^{r}\bar{a}(k-1)$  
$z_{i,r}(k|k-1)=\sum_{h=0}^{2n_{x}}W_{h}\varsigma_{h}(k|k-1)$  
$P_{i,r}(k|k-1)=\sum_{h=0}^{2n_{x}}W_{h}\Delta x_{i,h}(k|k-1)\Delta x_{i,h}^{T}(k|k-1)+Q^{r}(k-1)$ 
(2) update process noise covariance  $\sigma_{a}^{2}(k)=\left\{ \begin{array}{l} \frac{4-\pi }{\pi }{\left[a_{\max}-\bar{a}(k)\right]}^{2},\bar{a}(k)>0\\ \frac{4-\pi }{\pi }{\left[a_{-\max}-\bar{a}(k)\right]}^{2},\bar{a}(k)<0 \end{array}\right.$  
$Q^{r}(k)=2\alpha \sigma_{a}^{2}(k)q$ 
(3) define pseudo measurement matrix  $P_{i,r}^{xz}(k|k-1)=\sum_{h=0}^{2n_{x}}W_{h}\Delta x_{i,h}(k|k-1)\Delta z_{i,h}^{T}$  
$H_{i,r}^{T}(k)={\left(P_{i,r}(k|k-1)\right)}^{-1}P_{i,r}^{xz}(k|k-1)$ 
(4) update information matrix and information vector  $Y_{i,r}^{0}=\frac{1}{n}{\left(P_{i,r}(k|k-1)\right)}^{-1}+H_{i,r}^{T}(k)R_{i}^{-1}H_{i,r}(k)$  
$y_{i,r}^{0}=\frac{1}{n}{\left(P_{i,r}(k|k-1)\right)}^{-1}x_{i,r}(k|k-1)+H_{i,r}^{T}(k)R_{i}^{-1}(H_{i,r}(k)x_{i,r}(k|k-1)+z_{i}(k)-z_{i,r}(k|k-1))$ 
(5) perform consensus on $Y_{i,r}^{0}$ and $y_{i,r}^{0}$  
for l=1:L do   
(a) send $Y_{i,r}^{l-1}$ and $y_{i,r}^{l-1}$ to all neighbours $s\in N_{i}$   
(b) receive $Y_{s,r}^{l-1}$ and $y_{s,r}^{l-1}$ from all neighbours $s\in N_{i}$   
(c) update consensus terms:    $Y_{i,r}^{l}=Y_{i,r}^{l-1}+e_{i,s}(k)\sum_{s\in N_{i}}\left(Y_{s,r}^{l-1}-Y_{i,r}^{l-1}\right)$    
$y_{i,r}^{l}=y_{i,r}^{l-1}+e_{i,s}(k)\sum_{s\in N_{i}}\left(y_{s,r}^{l-1}-y_{i,r}^{l-1}\right)$ 
end for 
(6) compute a posteriori state estimate and covariance  $x_{i,r}(k|k)={\left(Y_{i,r}^{L}\right)}^{-1}y_{i,r}^{L}$  
$P_{i,r}(k|k)={\left(nY_{i,r}^{L}\right)}^{-1}$
**Step** **3.** Update mode probability $\Lambda_{i,r}(k)=\frac{1}{\sqrt{\left|2\pi S_{i,r}(k)\right|}}\exp[-\frac{1}{2}{\left(\nu_{i,r}(k)\right)}^{T}{\left(S_{i,r}(k)\right)}^{-1}\nu_{i,r}(k)]$ 
$u_{i,r}(k)=\frac{1}{C}\Lambda_{i,r}(k)\bar{C_{i,r}}$
**Step** **4.** Combination $x_{i}(k|k)=\sum_{r=1}^{m}x_{i,r}(k|k)u_{i,r}(k)$ 
$P_{i}(k|k)=\sum_{r=1}^{m}u_{i,r}(k)\{P_{i,r}(k|k)+[x_{i,r}(k|k)-x_{i}(k|k)]\cdot \;{\left[x_{i,r}(k|k)-x_{i}(k|k)\right]}^{T}\}$

### 4.3. Distributed Architecture

**Figure 2:**
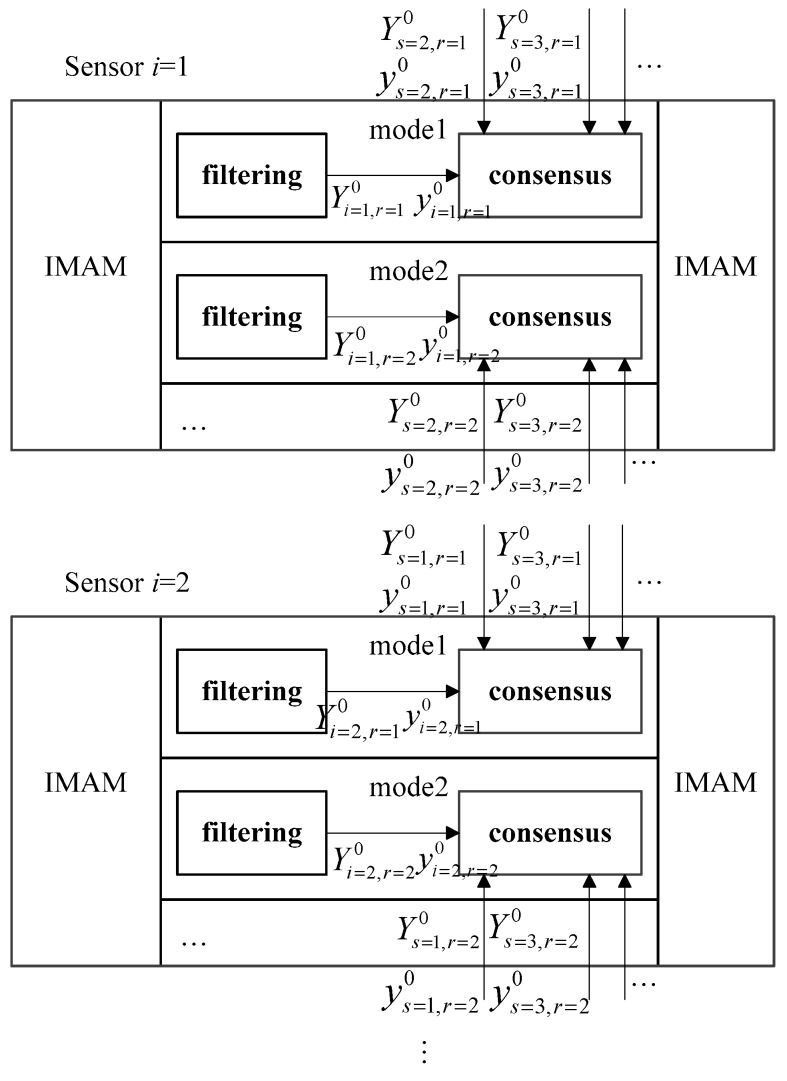
Structure of Interacting Multiple Adaptive Model-Unscented Information Consensus Filter.

In our algorithm, multiple sensors are used to track the target and they cooperate in the distributed architecture. Multiple fusion nodes process data from their own sensors and communicate with neighbor nodes. The data from other sensors can provide more information to improve upon the local results [30,31]. We perform distributed computing on each model separately so that more accurate model probabilities can be obtained. In this case, the model interaction of IMAM can play a greater role. The structure of the algorithm is shown in Figure 2. To improve the tracking effect and the agreement between sensors, distributed computing is based on a consensus protocol. 

## 5. Experimental Evaluation

### 5.1. Verification Experiment

To test the effectiveness of the proposed IMAM-UICF, a simple maneuvering target tracking problem is considered. The target motion switches between constant velocity model and constant acceleration model. A 6-D vector is chosen to express the state of target, including 2-D position(x,y), 2-D velocity$\left(\upsilon_{x},\upsilon_{y}\right)$ and 2-D accelerated velocity$\left(a_{x},a_{y}\right)$. The initial truth state of target is $x(0)={\left[10080,8000,-5.8,-11.6,0,0\right]}^{T}$. From 1s to 60s, target moves at constant velocity. From 61s to 110s, target moves at constant acceleration. From 111s to 170s, target moves at constant velocity. From 171s to 220s, target moves at constant acceleration. From 221s to 300s, target moves at constant velocity. The target motion can be described by constant velocity model

(29) $$x(k+1)=\left[\begin{array}{l} 1\;0\;T\;0\;0\;0\\ 0\;1\;0\;T\;0\;0\\ 0\;0\;1\;0\;0\;0\\ 0\;0\;0\;1\;0\;0\\ 0\;0\;0\;0\;0\;0\\ 0\;0\;0\;0\;0\;0 \end{array}\right]x(k)+\left[\begin{array}{l} 0.5T^{2}\;0\\ 0\;0.5T^{2}\\ T\;0\\ 0\;T\\ 0\;0\\ 0\;0 \end{array}\right]w_{CV}(k)$$

and constant acceleration model

(30) $$x(k+1)=\left[\begin{array}{l} 1\;0\;T\;0\;0.5T^{2}\;0\\ 0\;1\;0\;T\;0\;0.5T^{2}\\ 0\;0\;1\;0\;T\;0\\ 0\;0\;0\;1\;0\;T\\ 0\;0\;0\;0\;1\;0\\ 0\;0\;0\;0\;0\;1 \end{array}\right]x(k)+\left[\begin{array}{l} 0.5T^{2}\;0\\ 0\;0.5T^{2}\\ T\;0\\ 0\;T\\ 1\;0\\ 0\;1 \end{array}\right]w_{CA}(k)$$

where T=1s is the sampling interval.

A sensor network consisting of five sensor nodes is deployed to track the target. Sensors can measure distance and direction of the target according to the following equation

(31) $$z_{i}(k)=\left[\begin{array}{c} \sqrt{x^{2}+y^{2}}\\ \arctan(\frac{y}{x}) \end{array}\right]+r_{i}(k)\;i=1,2,\cdots ,5$$

The five sensors have different detection capabilities, so measurement noise covariance $R_{i}$ of sensor i is $\sqrt{i}\times diag([30^{2},{\left(0.3\pi /180\right)}^{2}])$. On the other hand, the five sensors are all static, so the position of sensor nodes is not considered. In addition, it is assumed that the target moves within all sensors’ detection range. 

Considering the limited energy and communication bandwidth in actual situations, the number of consensus iterations is set to L=8. The communication channels among five nodes can be expressed by adjacency matrix

(32) $$A=\left[\begin{array}{ccccc} 0 & 1 & 0 & 0 & 0\\ 1 & 0 & 1 & 0 & 0\\ 0 & 1 & 0 & 1 & 0\\ 0 & 0 & 1 & 0 & 1\\ 0 & 0 & 0 & 1 & 0 \end{array}\right]$$

***Model Parameters***

In IMAM, two CS models are chosen to estimate state of target and interact. The model parameters of five sensors are the same.

In model 1, the initial state $x^{1}(0|0)$ and covariance $P^{1}(0|0)$ of each sensor is equal, $x^{1}(0|0)={\left[10000,7900,-6,-10,0,0\right]}^{T}$ and $P^{1}(0|0)=diag([0.1,0.1,0.1,0.1,0.1,0.1])$. The maneuver frequency is α=0.1. The process noise variance matrix $Q^{1}$ is set to

(33) $$Q^{1}=\left[\begin{array}{l} 0.5T^{2}\;0\\ 0\;0.5T^{2}\\ T\;0\\ 0\;T\\ 1\;0\\ 0\;1 \end{array}\right]\left[0.04\;0.09\right]{\left[\begin{array}{l} 0.5T^{2}\;0\\ 0\;0.5T^{2}\\ T\;0\\ 0\;T\\ 1\;0\\ 0\;1 \end{array}\right]}^{T}$$

And state transition matrix

(34) $$F^{1}=\left[\begin{array}{cccccc} 1 & 0 & T & 0 & \left(\alpha T-1+e^{-\alpha T}\right)/\alpha^{2} & 0\\ 0 & 1 & 0 & T & 0 & \left(\alpha T-1+e^{-\alpha T}\right)/\alpha^{2}\\ 0 & 0 & 1 & 0 & \left(1-e^{-\alpha T}\right)/\alpha & 0\\ 0 & 0 & 0 & 1 & 0 & \left(1-e^{-\alpha T}\right)/\alpha \\ 0 & 0 & 0 & 0 & e^{-\alpha T} & 0\\ 0 & 0 & 0 & 0 & 0 & e^{-\alpha T} \end{array}\right]$$

In model 2, the initial state $x^{2}(0|0)$ and covariance $P^{2}(0|0)$ of each sensor is equal, $x^{2}(0|0)={\left[10000,7900,-6,-10,0,0\right]}^{T}$ and $P^{2}(0|0)=diag([0.1,0.1,0.1,0.1,0.1,0.1])$. The maneuver frequency is α=0.0045. The process noise variance matrix $Q^{2}$ is equal to (33), and state transition matrix $F^{2}$ is calculated as Equation (34).

The true motion of maneuvering target is uncertain. At each time step, the model probability is used to represent the likelihood that the target is moving in a certain model. The initial model probability is chosen to be $u_{1}(0)=0.5$ and $u_{2}(0)=0.5$. At the same time, the possibility of model conversion also needs to be considered. The transition probability is set to

(35) $$P=\left[\begin{array}{cc} 0.90 & 0.10\\ 0.10 & 0.90 \end{array}\right]$$

***Results and Analysis***

**Figure 3:**
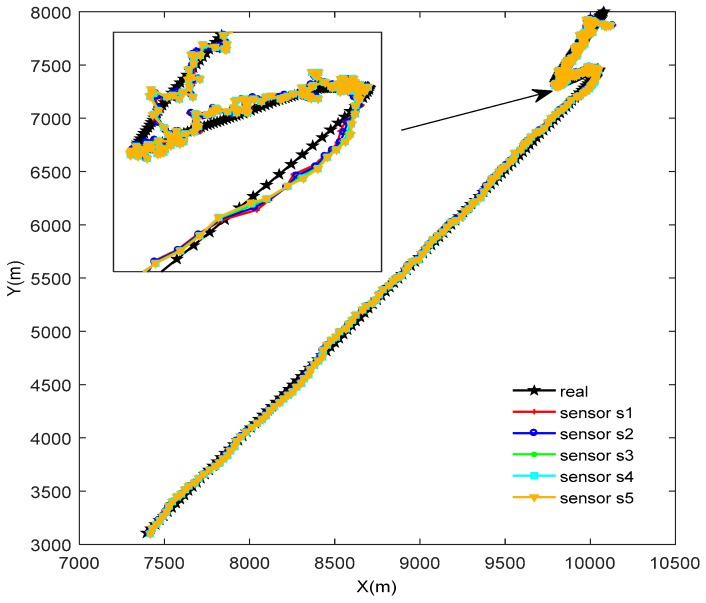
Real and estimated target trajectory.

**Figure 4:**
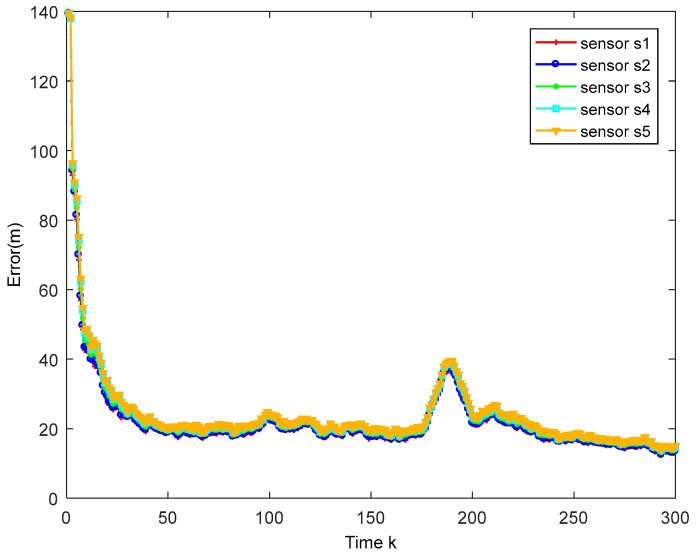
Position RMSE of different sensors.

**Figure 5:**
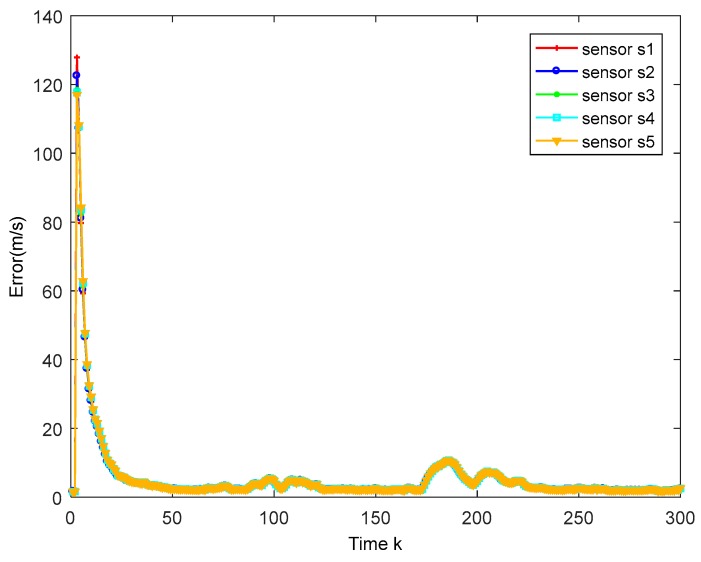
Velocity RMSE of different sensors.

**Figure 6:**
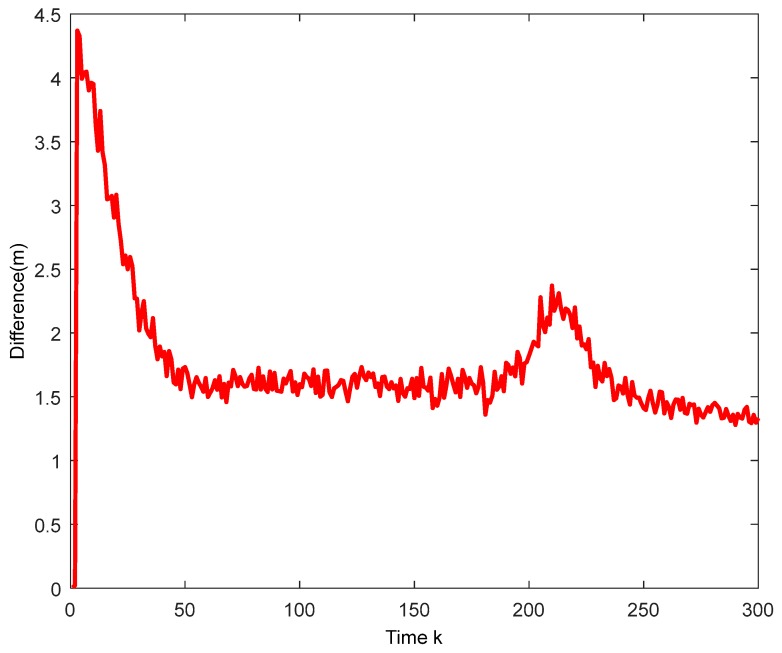
The disagreement between sensors.

The true trajectory and the estimated trajectory of multiple sensors are shown in Figure 3. The root mean square errors of all sensors’ estimated position and estimated velocity are shown in Figure 4 and Figure 5, in which each color corresponds to the root mean square error (RMSE) of each sensor. After a few times, the RMSEs converge to small values. Every sensor has a good track result. That proves our algorithm has an effective tracking performance. One advantage of the algorithm is that it can improve the consensus of multiple sensors’ estimation situations. The disagreement between all sensors is used to evaluate this performance. The result is shown in Figure 6.

### 5.2. Comparison Experiment

To validate the superiority of the proposed algorithm, the unscented transformation-based interacting multiple adaptive model filter (IMAM), interacting multiple adaptive model based on distributed unscented information filter (IMAM-DUIF) [5,32] and interacting multiple model-unscented information consensus filter (IMM-UICF) are chosen to be compared. In the IMAM method, we choose the best estimate throughout the whole sensor network to compare. IMAM-DUIF updates the state with neighboring information and there is no consensus protocol among them. In addition, IMM-UICF is the proposed method without adaptation.

In this scenario, the target moves with a complex maneuvering motion, including two strong maneuvers, which is a great challenge for sensors in the network. From 1 s to 110 s, the target dives and climbs, which are typical actions of military aircraft. From 111 s to 180 s, the target follows a snake maneuver. The details of the two maneuvers are shown in Table 1 and Table 2. Finally, from 181 s to 250 s, target moves at constant velocity. The initial truth state of the target is $x(0)={\left[12000,8000,-200,0,0,0\right]}^{T}$. To obtain more measurement from target, the sampling interval is set to T=0.5 s.

The constant velocity (CV) model and constant acceleration (CA) model have been described in Equations (29) and (30). The coordinate turn (CT) model is

(36) $$x(k+1)=\left[\begin{array}{ccccc} 1 & 0 & \frac{\sin(\omega T)}{\omega } & \frac{\cos(\omega T)-1}{\omega } & 0\\ 0 & 1 & \frac{1-\cos(\omega T)}{\omega } & \frac{\sin(\omega T)}{\omega } & 0\\ 0 & 0 & \cos(\omega T) & -\sin(\omega T) & 0\\ 0 & 0 & \sin(\omega T) & \cos(\omega T) & 0\\ 0 & 0 & 0 & 0 & 1 \end{array}\right]x(k)+\left[\begin{array}{l} 0.5T^{2}\;0\;0\\ 0\;0.5T^{2}\;0\\ T\;0\;0\\ 0\;T\;0\\ 0\;0\;1 \end{array}\right]w_{CT}(k)$$

The sensor network in verification experiment is also used to estimate the state of target in this experiment. The sensor parameters, communication channels and related assumptions are all the same. IMAM-DUIF, IMM-UICF and IMAM-UICF are implemented by the sensor network, while IMAM is implemented by the sensor 1. Sensor 1 has the best detection capabilities over the whole network, so the comparison can better illustrate that the track performance of sensor network is superior to single sensor.

***Model Parameters***

In IMAM, two CS models are chosen to estimate state of target and interact. The model parameters of the five sensors are also the same.

In model 1, the initial state $x^{1}(0|0)={\left[11600,7900,-180,0,0,0\right]}^{T}$ and covariance $P^{1}(0|0)=diag([0.1,0.1,0.1,0.1,0.1,0.1])$. The maneuver frequency is α=0.1. The state transition matrix $F^{1}$ is calculated as Equation (34). In addition, the process noise variance matrix $Q^{1}$ is set to

(37) $$Q^{1}=\left[\begin{array}{l} 0.5T^{2}\;0\\ 0\;0.5T^{2}\\ T\;0\\ 0\;T\\ 1\;0\\ 0\;1 \end{array}\right]\left[0.25\;0.25\right]{\left[\begin{array}{l} 0.5T^{2}\;0\\ 0\;0.5T^{2}\\ T\;0\\ 0\;T\\ 1\;0\\ 0\;1 \end{array}\right]}^{T}$$

In model 2, the initial state $x^{2}(0|0)={\left[11600,7900,-180,0,0,0\right]}^{T}$ and covariance $P^{2}(0|0)=diag([0.1,0.1,0.1,0.1,0.1,0.1])$. The maneuver frequency is α=0.0001. The state transition matrix $F^{2}$ is calculated as Equation (34). In addition, the process noise variance matrix $Q^{2}$ is equal to (37).

The initial model probability is chosen to be $u_{1}(0)=0.5$ and $u_{2}(0)=0.5$. In addition, the transition probability is set to

(38) $$P=\left[\begin{array}{cc} 0.90 & 0.10\\ 0.10 & 0.90 \end{array}\right]$$

As for IMM, a CV model and a CT model are chosen.

In the CV model, the initial state $x^{1}(0|0)={\left[11600,7900,-180,0\right]}^{T}$ and covariance $P^{1}(0|0)=diag([0.1,0.1,0.1,0.1])$. The state transition matrix $F^{1}$ can be seen in Equation (29), and the process noise variance matrix $Q^{1}$ is set to

(39) $$Q^{1}=\left[\begin{array}{l} 0.5T^{2}\;0\\ 0\;0.5T^{2}\\ T\;0\\ 0\;T \end{array}\right]\left[0.25\;0.25\right]{\left[\begin{array}{l} 0.5T^{2}\;0\\ 0\;0.5T^{2}\\ T\;0\\ 0\;T \end{array}\right]}^{T}$$

In CT model, the initial state $x^{2}(0|0)={\left[11600,7900,-180,0,0\right]}^{T}$ and covariance $P^{2}(0|0)=diag([0.1,0.1,0.1,0.1,0.1])$. The state transition matrix $F^{2}$ can be seen in Equation (36), and the process noise variance matrix $Q^{2}$ is set to

(40) $$Q^{2}=\left[\begin{array}{l} 0.5T^{2}\;0\;0\\ 0\;0.5T^{2}\;0\\ T\;0\;0\\ 0\;T\;0\\ 0\;0\;1 \end{array}\right]\left[0.25\;0.25\right]{\left[\begin{array}{l} 0.5T^{2}\;0\;0\\ 0\;0.5T^{2}\;0\\ T\;0\;0\\ 0\;T\;0\\ 0\;0\;1 \end{array}\right]}^{T}$$

***Results and Analysis***

**Figure 7:**
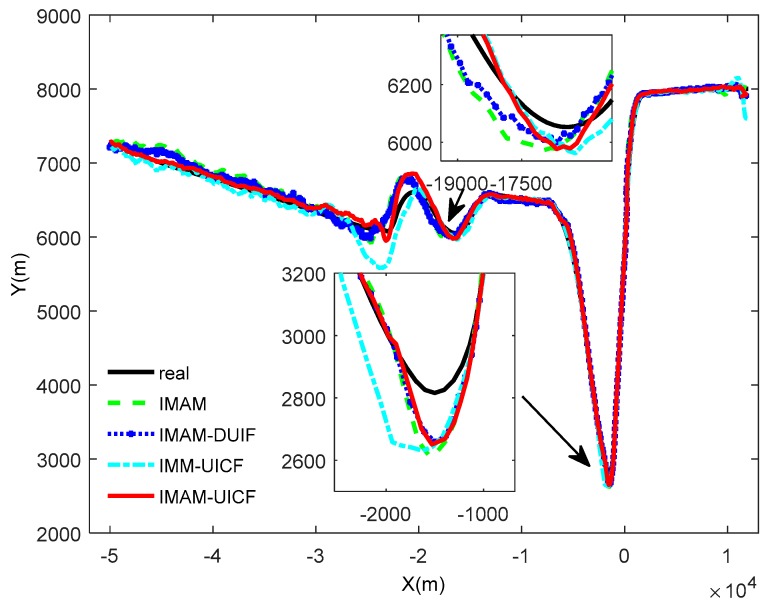
The trajectories of real and the four algorithms.

**Figure 8:**
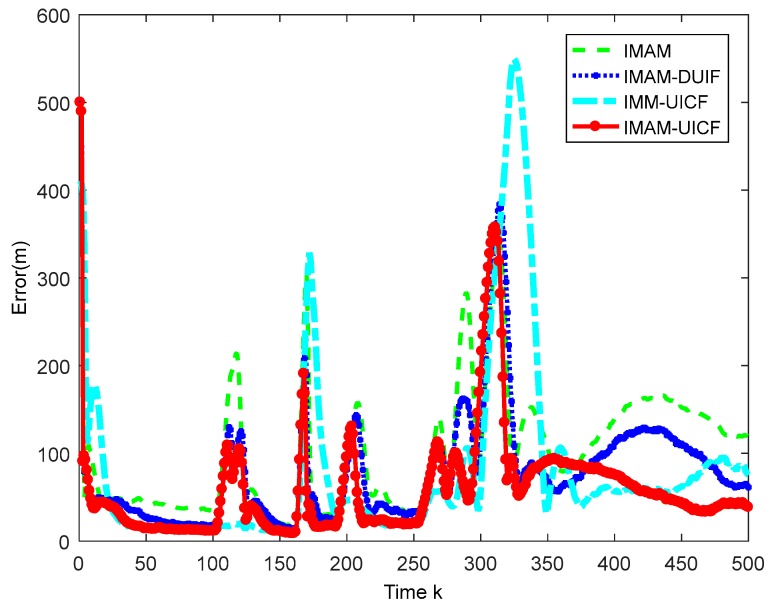
Position RMSE of different sensors.

**Figure 9:**
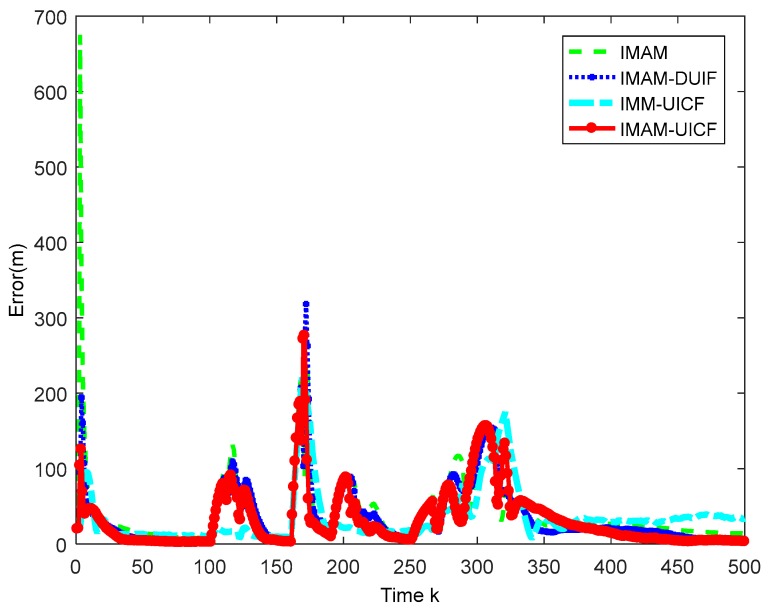
Velocity RMSE of different sensors.

**Figure 10:**
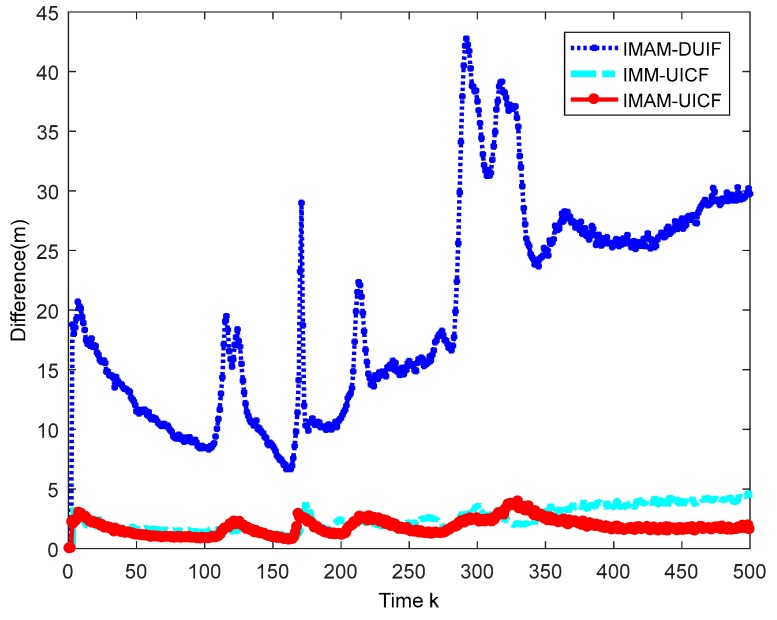
The disagreements of the three algorithms.

Considering that there are five sensors tracking the target, we only display the results of sensor 1 as a reference. In addition, sensor 1 is the chosen one in IMAM. The simulation results are shown in Figure 7, Figure 8, Figure 9 and Figure 10. Figure 7 shows that four methods are all effective in estimating the maneuvering target state. Figure 8 and Figure 9 are the root mean square position and velocity error of IMAM, IMAM-DUIF, IMM-UICF and IMAM-UICF, respectively. Even if the target has several strong maneuvers, they can get a relatively accurate estimate in a short time. The two figures demonstrate that all four methods can track the maneuvering target, but the best performance of the single sensor is poorer than that of the remaining three methods. Compared to IMAM-DUIF and IMM-UICF, IMAM-UICF is more accurate, especially for position estimation. For networked algorithms, it is essential to compare the disagreement of estimates among sensors throughout the sensor network, and the relatively high disagreement in the estimates goes against the purpose of tracking targets in a distributed way. Figure 10 shows that IMAM-UICF and IMM-UICF both have relatively low disagreements, while the estimates of sensors in IMAM-UICF are closer to each other than IMM-UICF. In addition, the two methods both perform better than IMAM-DUIF in terms of agreement.

To further validate the performance of IMAM-UICF, the accumulative root mean square error (ARMSE) of position estimates is defined in Equation (41).

(41) $$ARMSE_{\mathrm{pos}}=\sqrt{\frac{1}{\mathrm{MT}}\sum_{i=1}^{M}\sum_{k=1}^{T}\left({\left({\hat{x}}_{i}(k)-x_{i}(k)\right)}^{2}+{\left({\hat{y}}_{i}(k)-y_{i}(k)\right)}^{2}\right)}$$

where M is the number of Monte Carlo runs, and T is the total number of simulation steps.$\left({\hat{x}}_{i}(k),\;{\hat{y}}_{i}(k)\right)$ represents the estimated position in $i^{\mathrm{th}}$ simulation, and $\left(x_{i}(k),\;y_{i}(k)\right)$ represents the actual state. For simplicity, the definition of velocity ARMSE is not given here, which is similar to position ARMSE.

Table 3 shows that the two ARMSEs of IMM, IMAM-DUIF and IMM-UICF are larger than those of IMAM-UICF. In addition, the ARMSEs of IMAM-DUIF are smaller than those of IMAM, which shows the superiority of the sensor network. In particular, the position ARMSE of IMM-UICF is larger than that for the other methods. From the above comparisons, we can conclude that IMAM-UICF is superior among the three algorithms for maneuvering target tracking with respect to tracking accuracy and agreement among sensors. The time in Table 3 represents the computational cost of four methods. They are the calculation time of a single sensor at a sampling time. IMAM takes the shortest time, because the sensors do not need to communicate with neighbor sensors. Due to the iterative operations of consensus protocol, IMAM-UICF takes more time than IMAM-DUIF. In addition, the cost of IMAM-UICF is higher than IMM-UICF because of model adaptation.

### 5.3. Experiment with Varying Numbers of Sensors n

**Figure 11:**
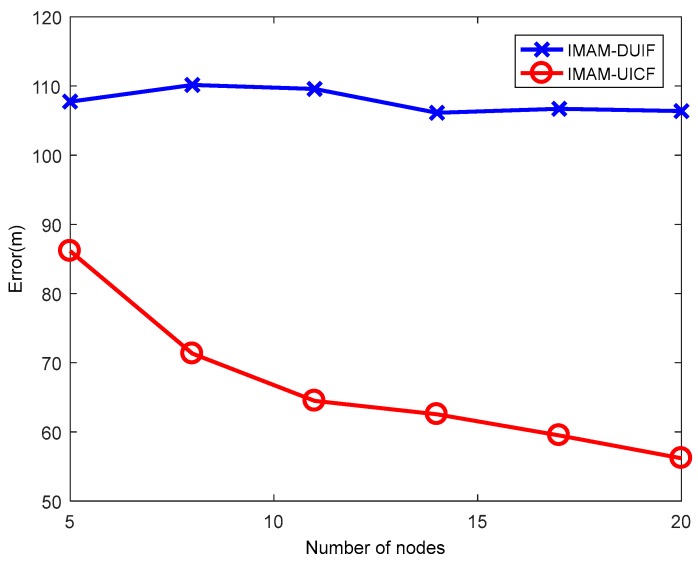
The position ARMSE of the two algorithms with varying number of sensors.

**Figure 12:**
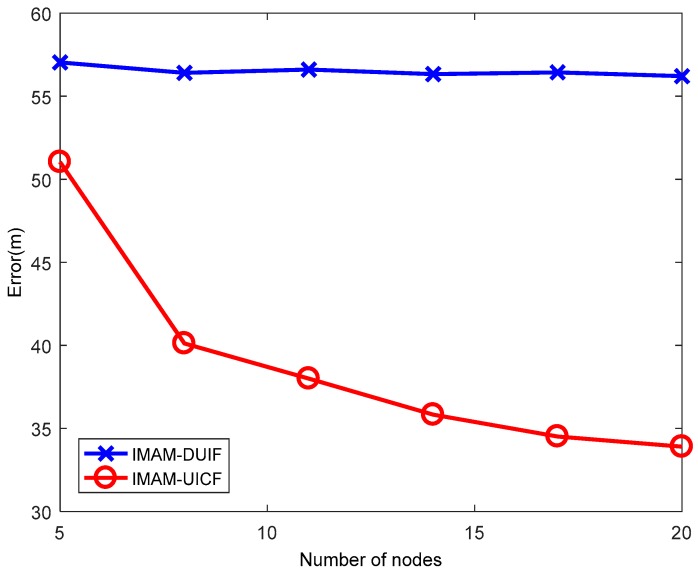
The velocity ARMSE of the two algorithms with varying number of sensors.

This experiment aims to verify the advantages of the consensus protocol. In this experiment, n is varied from 5 to 20 at increments of 3. The results are shown in Figure 11 and Figure 12. In the figures, when n is increased, the error of IMAM-UICF decreases, while the error of IMAM-DUIF shows almost no change. In the distributed architecture, each sensor communicates with its neighbor nodes. So each sensor can only get part of the information in the network, obtaining a local estimate. In IMAM-UICF, using consensus iteration, each node can indirectly fuse information from other nodes (those are not its neighbors). Thus, as the number of sensors is increased, the estimation results of IMAM-UICF are closer to the optimal global estimate.

### 5.4. Experiment of Varying Measurement Noise r_i_

**Figure 13:**
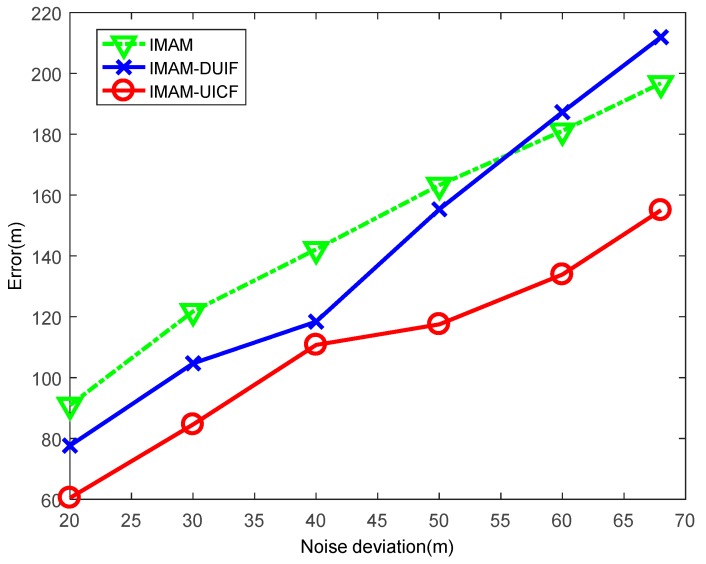
The position ARMSE of the three algorithms with varying measurement noise.

**Figure 14:**
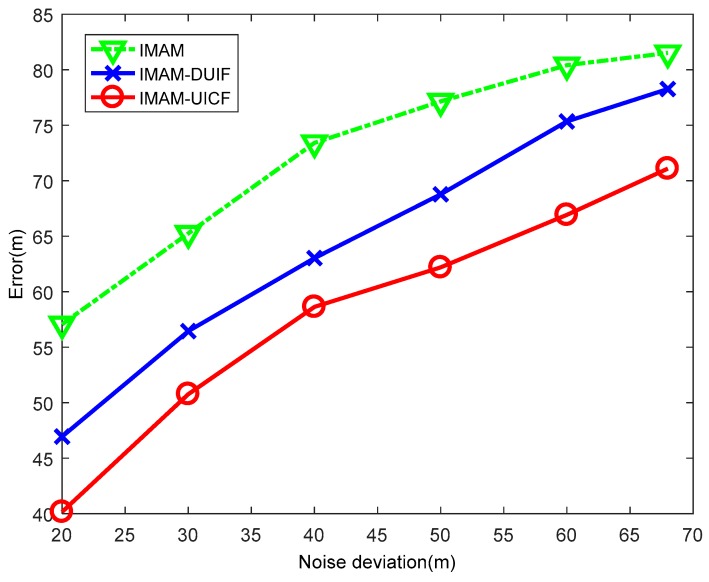
The velocity ARMSE of the three algorithms with varying measurement noise.

This experiment aims to compare the performance of the three algorithms for varying measurement noise $r_{i}$. In this experiment, the standard deviation of $r_{i}$ is varied from ${\left[20,(0.2\pi /180)\right]}^{T}$ to ${\left[68,(0.68\pi /180)\right]}^{T}$. The results are shown in Figure 13 and Figure 14. In the figures, when the standard deviation of noise is small, the position ARMSE and velocity ARMSE of IMAM-UICF are the lowest among the 3 algorithms. With increased standard deviation of noise, the estimation accuracies of three methods decreases. When the standard deviation of noise is large, IMAM-DUIF has a higher position error than IMAM, and IMAM-UICF still has a better estimate in position and velocity. From the results, it can be seen that IMAM-UICF outperforms the other two methods for different measurements.

## 6. Conclusions

In this paper, an adaptive information-weighted consensus filter for maneuvering target tracking problem is proposed. Combined with adaptive current statistical models to handle the dynamic uncertainty, the true acceleration of a maneuvering target is more accurately estimated. The nonlinearity problem is solved using unscented transformation, which avoids the linearization errors of Taylor series expansions in the extended Kalman filter, and reduces the computational cost. The information-weighted consensus protocol and adaptive interacting multiple models are applied to improve the estimation accuracy and unify the estimates of maneuvering targets in the entire sensor network, which makes a shared situation picture in each sensor node. The simulation results illustrate that the proposed IMAM-UICF outperforms IMAM, IMAM-DUIF and IMM-UICF with respect to estimation accuracy and agreement of estimates in the entire sensor network.

## Figures and Tables

**Table 1:** Diving and climbing.

Time (s)	1–51	51–61	61–81	81-–85	85–87	87–91	91–96	96–110
Model	CV	CT	CV	CT	CA	CA	CA	CT
$a_{x}$	0	ω=0.12	0	ω=−0.4	0	0	−5	ω=0.06
$a_{y}$	0		0		20	15	10	

Where ω=0.12 is the angular velocity.

**Table 2:** Snake maneuver.

Time(s)	110–126	126–134	134–145	145–157	157–161	161–163	163–178	178–180
Model	CV	CA	CA	CA	CA	CA	CA	CV
$a_{x}$	0	5	−8	10	0	−10	−5	0
$a_{y}$	0	−10	18	−20	30	−8	0	0

**Table 3:** Performance comparison of different algorithms.

Algorithms	Position ARMSE (m)	Velocity ARMSE (m/s)	Time (s)
IMAM	124.0424	65.6603	0.001400000
IMAM-DUIF	103.8004	55.8458	0.001557773
IMM-UICF	137.0088	51.4543	0.001606565
IMAM-UICF	86.2178	50.7479	0.001806565
